# The comparative burden of brain and central nervous system cancers from 1990 to 2019 between China and the United States and predicting the future burden

**DOI:** 10.3389/fpubh.2022.1018836

**Published:** 2022-10-21

**Authors:** Jiajia Huang, Hanmei Li, Hualing Yan, Fen-Xiang Li, Mai Tang, Da-Lin Lu

**Affiliations:** Department of Epidemiology, School of Medicine, Jinan University, Guangzhou, China

**Keywords:** brain and central nervous system cancers, burden, age-period-cohort model, relative risk, prediction

## Abstract

**Background:**

Brain and central nervous system (CNS) cancers represent a major source of cancer burden in China and the United States. Comparing the two countries' epidemiological features for brain and CNS cancers can help plan interventions and draw lessons.

**Methods:**

Data were extracted from the Global Burden of Disease repository. The average annual percentage change (AAPC) and relative risks of cancer burdens were calculated using joinpoint regression analysis and age-period-cohort (APC) models, respectively. Moreover, a Bayesian APC model was employed to predict the disease burden over the next decade.

**Results:**

From 1990 to 2019, the number of incidences, deaths, and disability-adjusted life-years (DALYs) increased in China and the US, with a larger increase in China. Age-standardized incidence rates in China and the United States have shown an increasing trend over the past three decades, with AAPCs of 0.84 and 0.16%, respectively. However, the rates of age-standardized mortality and age-standardized DALYs decreased in both countries, with a greater decrease in China. Overall, age trends in cancer burden were similar for males and females, with two peaks in the childhood and elderly groups, respectively. The period and cohort effects on incidence showed an overall increasing trend in China and limited change in the US. However, the period effects for mortality and DALY were decreasing in both countries, while the cohort effects tended to increase and then decrease. Moreover, we predicted that the cancer burdens would continue to rise in China over the next decade.

**Conclusion:**

The burden of brain and CNS cancers is substantial and will continue to increase in China. Comprehensive policy and control measures need to be implemented to reduce the burden.

## Introduction

Brain and central nervous system (CNS) cancers comprise a group of rare and heterogeneous tumors originating in the brain and surrounding structures (such as meninges, spinal cord, and cranial nerves) (1). Brain and CNS tumors were relatively rare worldwide, accounting for ~1.6% of all cancers in adults (2). However, primary malignant brain and CNS cancers have a poor prognosis and directly affect neurological function with headache, vision loss, seizures, speech impairment, and paralysis (3–5). Moreover, even benign tumors can be lethal due to their site in the brain, with high mortality and disability rates that severely impair the independent function of patients (6). Approximately 308,102 new cases and 251,329 brain and CNS tumor deaths were diagnosed globally in 2020 (2). The incidence of brain and central nervous system tumors has increased in several regions since the standardization of tumor registration and the improvement of diagnostic techniques (4, 7, 8).

There are significant geographical differences in the epidemiology of brain and central nervous system cancers, with the highest incident numbers in East Asia, especially in China (4). A global systematic study on brain and CNS tumors observed that the top two countries with the highest number of incident cases were China and the United States (4). World Health Organization (WHO) reported 79,575 newly diagnosed brain and CNS tumor cases and 65,204 deaths in China in 2020, placing a substantial disease burden on China (9). The most recent statistical report of The Central Brain Tumor Registry of the United States (CBTRUS) showed that 432,693 diagnosed cases and 83,029 deaths were attributed to malignant brain and CNS tumors between 2014 and 2018 (10). The health of the population in China and the United States is threatened significantly by brain and CNS cancers. Knowing the epidemiological features of brain and CNS cancers is critical for preventing and managing cancer.

The CBTRUS statistical report in the US is the largest histology-specific summary of brain and CNS tumors in the world, which regularly releases detailed reports on brain tumor epidemiologic trends, histologic type distribution, and associated factors over the past 5 years (10). However, the Chinese National Brain Tumor Registry was established in 2018, and data collection is in progress (11). The relevant studies on the burden of brain and CNS cancers were scarce or incomplete in China. Higher incidence and mortality of brain cancer in countries with higher Human Development Index (HDI) levels than in countries with lower HDI levels (12). The United States is a developed country with the highest level of economic development, and China is the largest developing country in the world and is experiencing rapid economic development (13). Therefore, a comparative analysis of the burden of brain and CNS cancers between China and the US is vital for formulating long-term prevention strategies to improve public health.

Previous studies have focused on temporal trends in brain cancer burden without investigating the effects of age, period, and cohort on brain tumor burden, there was no comparative study on epidemiological trends and impact factors (e.g., age, period, and cohort) between China and the US (4, 14, 15). The Global Burden of Disease (GBD) study included extensive epidemiological data on the disease since 1990, allowing us to compare the burden of brain tumors in China and the US. The present study compared trends in the burden of brain and CNS cancers in China and the US from 1990 to 2019 and explored their relationship with age, period, and birth cohort based on data from GBD 2019 for the first time. Moreover, we predicted the disease burden from 2020 to 2030 for the two countries based on the Bayesian age-period-cohort (BAPC) model. Our results may contribute to enhancing the understanding of the epidemiological characteristics and influencing factors of brain and CNS cancers in China, and provide evidence for the prevention and management of brain and CNS cancers in China by comparing with the US.

## Methods

### Study data

The data on the annual number of brain and CNS cancers related incident cases, deaths, and DALYs by sex and age from 1990 to 2019 were extracted from the official website of the GBD 2019 Study. The GBD 2019 is a large international collaborative project providing information on the global burden of more than 300 diseases, available from the Institute for Health Metrics and Evaluation (IHME) for free (http://ghdx.healthdata.org/gbd-results-tool). The GBD data in China were primarily derived from the Chinese Center for Disease Control and Prevention, the China Cancer Registry, the Disease Surveillance Point system, national surveys, and published studies (16). The data sources in the US were primarily from disease surveillance systems, vital registrations, systematic reviews, and surveys (15). The detailed source of data can be found in the source tool of Global Health Data Exchanges (https://ghdx.healthdata.org/gbd-2019/data-input-sources) (17). Previous studies have described detailed methods for GBD studies (17, 18). All cancers coded C70.0–C72.9 (C70, malignant neoplasm of meninges; C71, malignant neoplasm of brain; C72, malignant neoplasm of spinal cord, cranial nerves, and other parts of the CNS) are classified as brain and CNS cancers in the GBD based on the 10th revision of the International Classification of Diseases (ICD) (4). All estimates computed in GBD from 1990 to 2019 were carried out 1,000 times at the draw level to account for uncertainty from input data, data adjustments, and model selection. The bounds of the 95% uncertainty intervals (UIs) were based on sequential percentages of 2.5 and 9.75 of 1,000 draws of the uncertainty distribution (17).

Population data between 1990 and 2019 were extracted from the GBD repository (https://ghdx.healthdata.org/record/ihme-data/gbd-2019-population-estimates-1950-2019). To project the future burden of brain and CNS cancers, we extracted the population forecast data for China and the US during 2020–2030 from the GBD repository (https://ghdx.healthdata.org/record/ihme-data/global-population-forecasts-2017-2100). These population data were estimated based on the data from censuses and population registry location years in the respective country (18).

### Statistical analysis

In this study, we conducted a three-stage analysis. First, we described the incidence, mortality, and DALYs of brain and CNS cancers and burden changes from 1990 to 2019 in China and the US by sex. To reflect changes in the burden of disease caused by brain and CNS cancers, we employed the joinpoint regression model (version 4.9.0.1, Joinpoint, IMS, Calverton, MD, USA) to calculate the average annual percentage change (AAPC) for quantifying temporal trends for the incidence, mortality, and DALYs of brain and CNS cancers from 1990 to 2019 in China and the US. We also calculated the 95% confidence interval (CI) of AAPC in the regression model. The AAPC estimate and the lower bound of 95% CI >0 indicates that the age-standardized rate increases, whereas the AAPC estimate and the upper bound of 95% CI <0 indicate that the age-standardized rate decreases. When the 95% CI of an AAPC includes zero implies that the age-standardized rate is stable over time.

Second, we applied the age-period-cohort (APC) model to estimate the effects of age, period, and cohort on trends in the incidence, mortality, and DALYs rates (19, 20). The age effect reflects the biological and social processes of aging, which represents different risks of the related outcomes in different age groups (19, 21). The period effect represents the effect of influencing factors (such as social, economic, and cultural factors) on the related outcomes of all age groups (19, 21). The cohort effect represents variations across groups of individuals born during the same period and changes in different lifestyles (19, 21). For conducting APC analysis, the data were categorized into 17 consecutive 5-year age groups from 0–4 years to 80–84 years old, 6 consecutive 5-year periods from 1990–1994 to 2015–2019, and 22 consecutive birth cohorts from 1910–1914 to 2015–2019. Our analysis calculated the cohort RRs using the median of the birth cohort (1960–1964) as the reference and the period RRs applying the median of the study period (2000–2004) as the reference. We employed the APC analyses through the age-period-cohort Web Tool from the Division of Cancer Epidemiology and Genetics at the US National Cancer Institute (http://analysistools.nci.nih.gov/apc/) (22). The Wald Chi-Square tests were employed to test the significance of the parameters. *P*-value < 0.05 (two-sided) was considered statistically significant.

Finally, we used the BAPC model to predict the number and rate of the disease burden attributable to brain and CNS cancers from 2020 to 2030. The Bayesian inference in APC models smooths the prior information on age, period, and cohort effects based on a second-order random walk and predicts posterior rates (23). Several studies evaluating different prediction models found that the BAPC model had the best prediction performance (24–26). The BAPC model is based on an integrated nested Laplacian approximation to approximate marginal posterior distributions, avoiding some of the mixing and convergence problems introduced by the Markov Chain Monte Carlo sampling technique traditionally used for Bayesian methods (26). To evaluate the performance of the BAPC model, we conducted a comparative study between the BAPC and the Nordpred model (27). We divided the data into two intervals (1990–2014 and 2015–2019). The data from 1990 to 2014 were used to train Nordpred and BAPC models to predict the burden of brain and CNS cancers for 2015–2019 and compare them with observed values for the same period. The prediction error rate was employed to assess the model performance. The error rate was calculated as ŷ−*y*/*y*, where ŷ and y denote the prediction values and the observational values, respectively. Because the BAPC model had a relatively lower error rate (Supplementary Figure 1), we used it to predict the burden of brain and CNS cancers during 2020–2030. The predicted incidence, mortality, and DALYs rate were standardized based on the GBD world population age standard (18). The BAPC and integrated nested Laplacian approximation (INLA) packages of R program (version 4.1.0) were applied to conduct the forecast.

## Results

### The disease burden related to brain and CNS cancers in China and the US

In China, the number of incident cases, deaths, and DALYs of brain and CNS cancers increased from 45.85 (95% UI 35.18–61.35) thousand to 94.69 (95% UI 73.4–114.09) thousand, 37.97 (95% UI 29.1–50.21) thousand to 63.53 (95% UI 47.79–76.95) thousand, and 1769.66 (95% UI 1286.81–2432.38) thousand to 2053.42 (95% UI 1584.34–2524.97) thousand from 1990 to 2019, respectively. Simultaneously, the number of incident cases, deaths, and DALYs of brain and CNS cancers increased from 17.08 (95% UI 14.78–20.49) thousand to 28.02 (95% UI 21.37–33.11) thousand, 12.67 (95% UI 10.98–15.22) thousand to 20.46 (95% UI 15.7–22.01) thousand, and 406.14 (95% UI 351.31–484.92) thousand to 565.06 (95% UI 459.09–619.53) thousand in the US, respectively (Table 1).

**Table 1:** The number and the age-standardized rates of incidence, deaths, and DALYs for brain and CNS cancers in China and the US and the average annual percentage change from 1990 to 2019.

	**China**					**The United States**				
	**1990**		**2019**		**1990–2019**	**1990**		**2019**		**1990–2019**
	**N × 10^3^ (95% UI)**	**ASR/10^5^ (95% UI)**	**N × 10^3^ (95% UI)**	**ASR/10^5^ (95% UI)**	**AAPC (95% CI)**	**N × 10^3^ (95% UI)**	**ASR/10^5^ (95% UI)**	**N × 10^3^ (95% UI)**	**ASR/10^5^ (95% UI)**	**AAPC (95% CI)**
**Incidence**										
Both	45.85 (35.18, 61.35)	4.45 (3.47, 5.94)	94.69 (73.4, 114.09)	5.69 (4.36, 6.78)	0.84 (0.63, 1.05)	17.08 (14.78, 20.49)	6.03 (5.23, 7.26)	28.02 (21.37, 33.11)	6.3 (4.94, 7.46)	0.16 (0.03, 0.29)
Female	20.76 (13.75, 28.16)	4.09 (2.75, 5.55)	47.62 (34.83, 62.14)	5.83 (4.30, 7.77)	1.2 (0.91, 1.50)	7.8 (6.2, 10.03)	5.12 (4.07, 6.54)	12.34 (8.27, 15.51)	5.32 (3.68, 6.65)	0.14 (0.00, 0.29)
Male	25.09 (16.49, 37.77)	4.84 (3.21, 7.23)	47.07 (30.53, 61.74)	5.64 (3.61, 7.33)	0.53 (0.33, 0.72)	9.28 (7.45, 11.21)	7.10 (5.73, 8.61)	15.68 (11.53, 19.54)	7.39 (5.55, 9.24)	0.15 (0.01, 0.29)
**Death**										
Both	37.97 (29.1, 50.21)	3.87 (3.04, 5.10)	63.53 (47.79, 76.95)	3.5 (2.62, 4.21)	−0.37 (−0.5, −0.23)	12.67 (10.98, 15.22)	4.29 (3.73, 5.16)	20.46 (15.7, 22.01)	4.12 (3.24, 4.45)	−0.15 (−0.25, −0.04)
Female	16.71 (11.63, 22.37)	3.44 (2.44, 4.58)	27.88 (20.2, 36.13)	3.01 (2.19, 3.88)	−0.47 (−0.57, −0.37)	5.76 (4.60, 7.48)	3.53 (2.81, 4.54)	8.85 (6.03, 10.01)	3.34 (2.35, 3.85)	−0.2 (−0.28, −0.12)
Male	21.26 (14.50, 31.89)	4.35 (3.02, 6.46)	35.65 (22.01, 47.44)	4.05 (2.53, 5.33)	−0.24 (−0.36, −0.13)	6.91 (5.49, 8.31)	5.2 (4.13, 6.26)	11.61 (8.64, 13.3)	5.01 (3.79, 5.83)	−0.12 (−0.24, −0.01)
**DALYs**										
Both	1769.66 (1286.81, 2432.38)	161.29 (118.00, 220.25)	2053.42 (1584.34, 2524.97)	126.24 (96.01, 154.8)	−0.81 (−0.91, −0.70)	406.14 (351.31, 484.92)	149.44 (129.29, 178.22)	565.06 (459.09, 619.53)	133.86 (110.51, 148.95)	−0.39 (−0.51, −0.28)
Female	763.83 (493.38, 1042.82)	144.01 (93.80, 195.96)	875.14 (649.70, 1156.06)	109.48 (80.78, 144.7)	−0.92 (−1.14, −0.69)	176.15 (139.56, 225.08)	123.68 (97.95, 158.36)	237.41 (169.65, 279.99)	109.61 (80.27, 131.75)	−0.42 (−0.49, −0.34)
Male	1005.83 (655.96, 1568.96)	178.2 (117.52, 276.17)	1178.28 (743.32, 1566.66)	143.02 (89.74, 189)	−0.76 (−0.89, −0.63)	229.99 (183.66, 274.61)	178.03 (141.70, 213.30)	327.64 (256.69, 394.38)	159.77 (127.72, 195.13)	−0.36 (−0.45, −0.27)

CNS, central nervous system; DALYs, disability-adjusted life years; UI, uncertainty interval; CI, confidence interval; AAPC, average annual percentage change.

**Figure 1:**
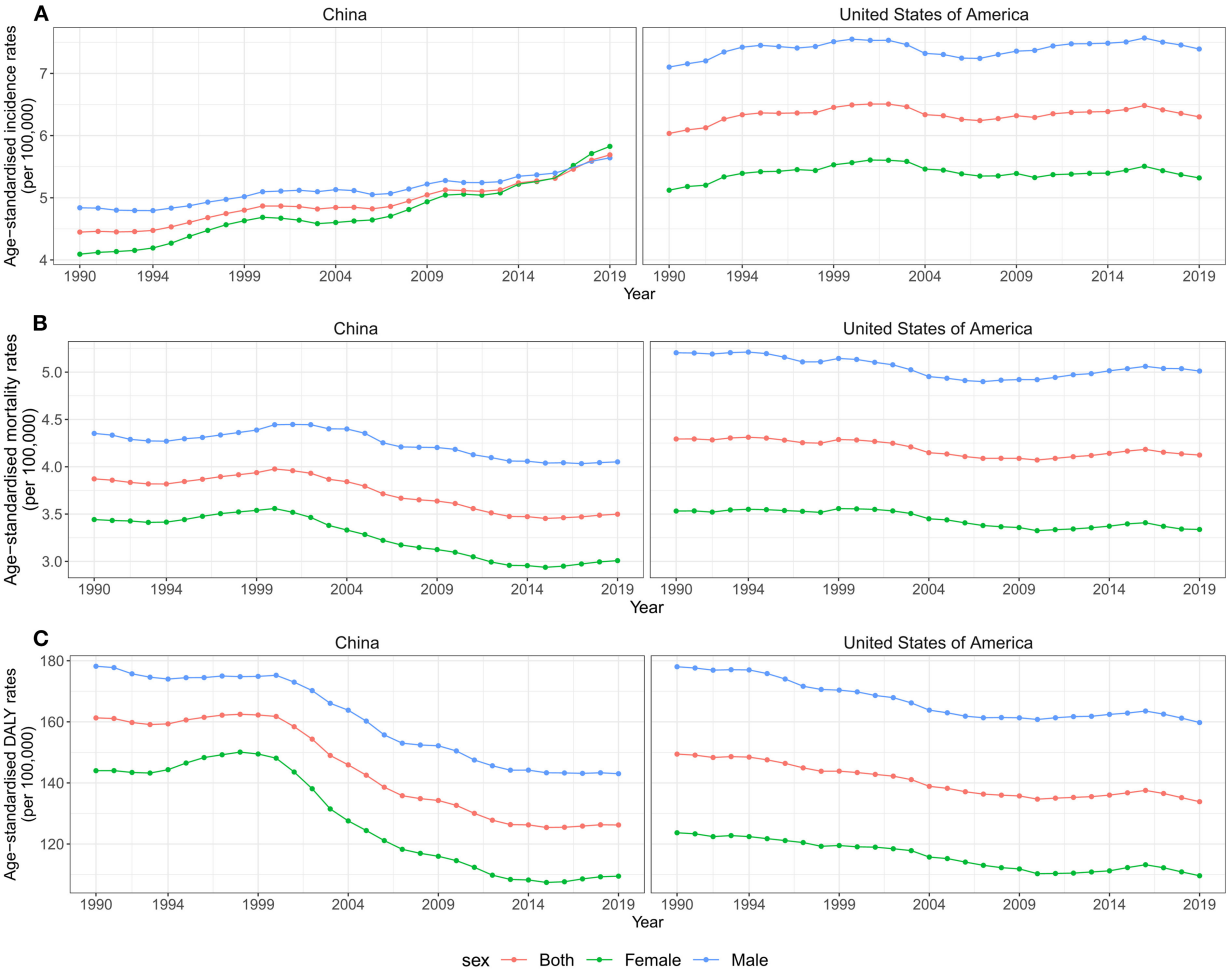
Trends for ASIR, ASMR, and ASDR of brain and CNS cancer by sex in China and the US from 1990 to 2019. **(A)** ASIR of brain and CNS cancers for both sexes in China and the US from 1990 to 2019. **(B)** ASMR of brain and CNS cancers for both sexes in China and the US from 1990 to 2019. **(C)** ASDR of brain and CNS cancers for both sexes in China and the US from 1990 to 2019. ASIR, age-standardized incidence rate; ASMR, age-standardized mortality rate; ASDR, age-standardized disability-adjusted life years rate; CNS, central nervous system.

Although the number of incidences, deaths, and DALYs was higher and increased substantially in China than in the United States between 1990 and 2019, ASIR and age-standardized mortality rates (ASMR) were consistently higher in the US. Overall, there was a continuously increasing trend for ASIR in both countries. In China, the ASIR increased 0.84% (95% CI 0.63, 1.05%) per year from 1990 to 2019, while the US increased ~0.16% (95% CI 0.03, 0.29%) per year. In contrast to ASIR, ASMR and age-standardized DALY rates (ASDR) decreased from 1990 to 2019 in both countries, with higher declines in China (Table 1, Figure 1).

Furthermore, we analyzed the changing trends of disease burden in both gender populations. As shown in Figure 1, the ASMR and the ASDR were higher in males than females in both countries between 1990 and 2019. The incident cases and ASIR were higher in males than females in China in 1990, while the reverse was observed in 2017. Overall, the ASIR attributable to brain and CNS cancers increased in both sexes from 1990 to 2019, especially for female patients in China, with an AAPC of 1.2% (95%CI 0.91, 1.5%). However, the ASMR and the ASDR in both sexes showed a downward trend in both countries during the study period (Table 1, Figure 1).

**Figure 2:**
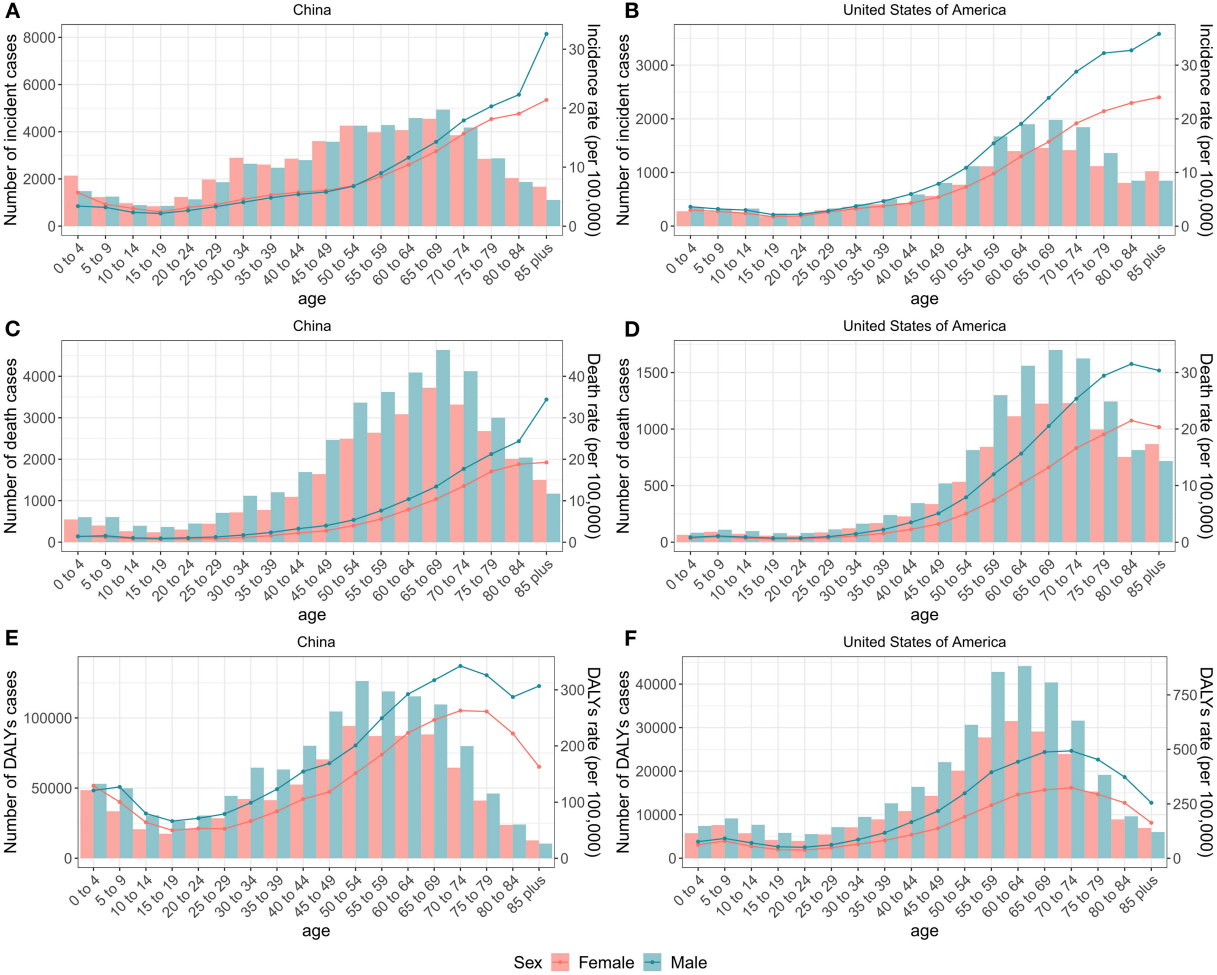
Numbers and rates of incidence, death, and DALYs of brain and CNS cancers for each age group by sex in China and the US, 2019. The number and rate of incidence of brain and CNS cancers for each age group by sex in China **(A)** and the US **(B)**. The number and rate of deaths of brain and CNS cancers for each age group by sex in China **(C)** and the US **(D)**. The number and rate of DALYs of brain and CNS cancers for each age group by sex in China **(E)** and the US **(F)**. Lines indicate rate and bars indicate absolute number. DALYs, disability-adjusted life-years; CNS, central nervous system.

Figure 2 presents the number and rate of incidence, mortality, and DALYs attributed to brain and CNS cancers in China and the US by age and sex in 2019. Overall, trends were similar for males and females, with the age distribution tending to be bimodal in both countries. We observed a small peak mainly in the children group and a large peak mainly in the elderly group.

### The age, period, and cohort effects on the burden of brain and CNS cancers

**Figure 3:**
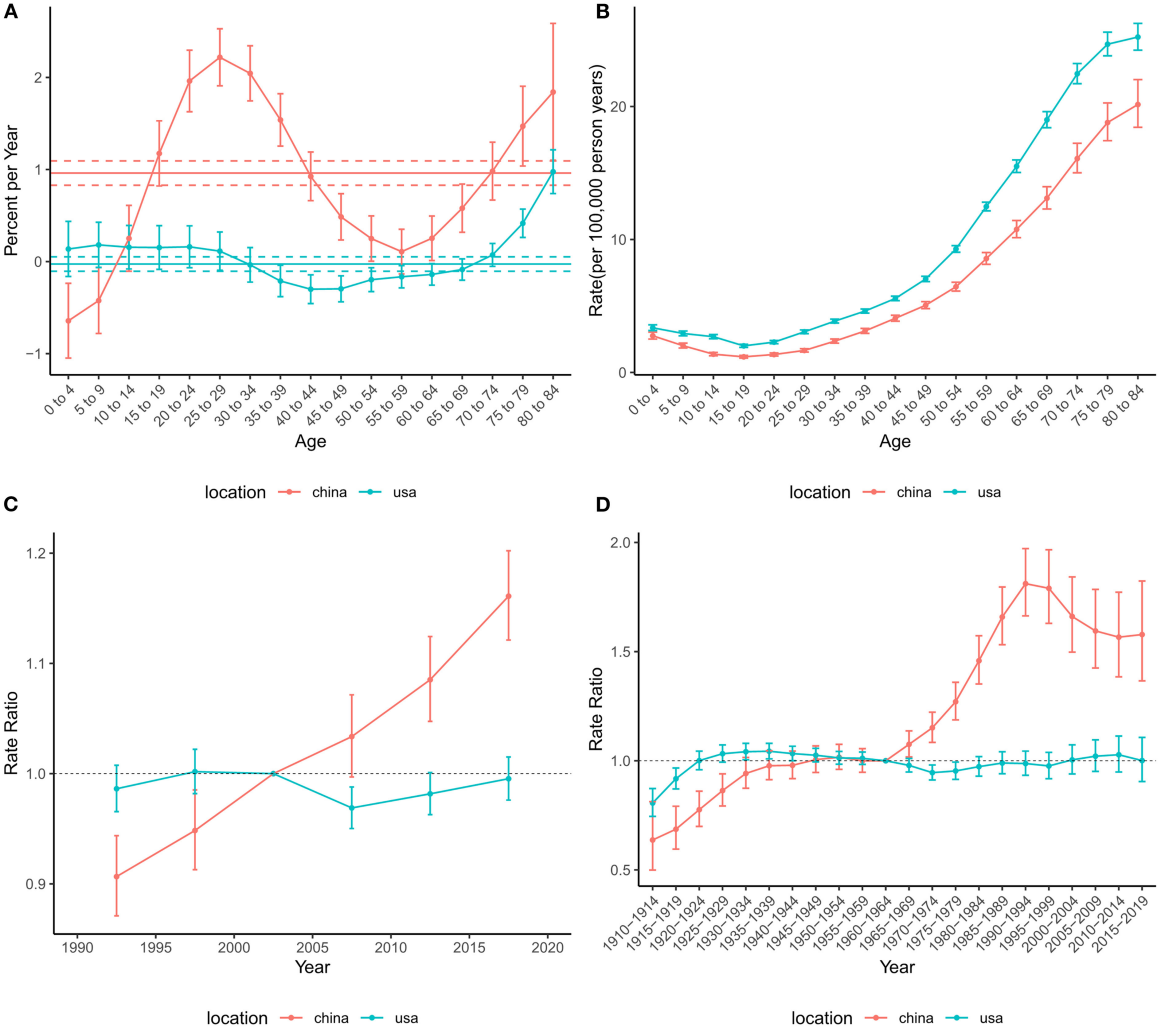
Age-period-cohort effects on the incidence rate of brain and CNS cancers in China and the US. **(A)** Net drift and local drift values for brain and CNS cancers incidence rate in China and the US. **(B)** Longitudinal age curves for brain and CNS cancers incidence in China and the US. **(C)** Period effects on the brain and CNS cancers incidence rate in China and the US relative to the reference period (2000–2004). **(D)** Cohort effects on the brain and CNS cancers incidence rate in China and the US relative to the reference cohort (1960–1964). Vertical solid bars represent the 95% confidence intervals. CNS, central nervous system.

The effects of age, period, and cohort on the incidence of brain and CNS cancers in China and globally are shown in Figure 3. The annual percentage changes (net drift) were 0.96% (95% CI: 0.83 to 1.09%) and −0.03% (95% CI: −0.11% to 0.05%) for China and the US, respectively. Moreover, we found positive values of local drift in all Chinese people over 14 years of age, indicating an increasing incidence in people over 14 years of age. The age effect on incidence tended to decrease and then increase in both China and the US, with two peaks in the childhood and elderly groups, respectively. The period effect revealed rate ratio (RR) of incidence increased in China during the study period, while little change was observed in the United States. Overall, the cohort effect in China and the US showed an upwards trend. The cohort effect for incidence increased before 1990–1994 and then decreased in China, while the cohort risk of incidence in the US changed unremarkably after 1925–1929.

**Figure 4:**
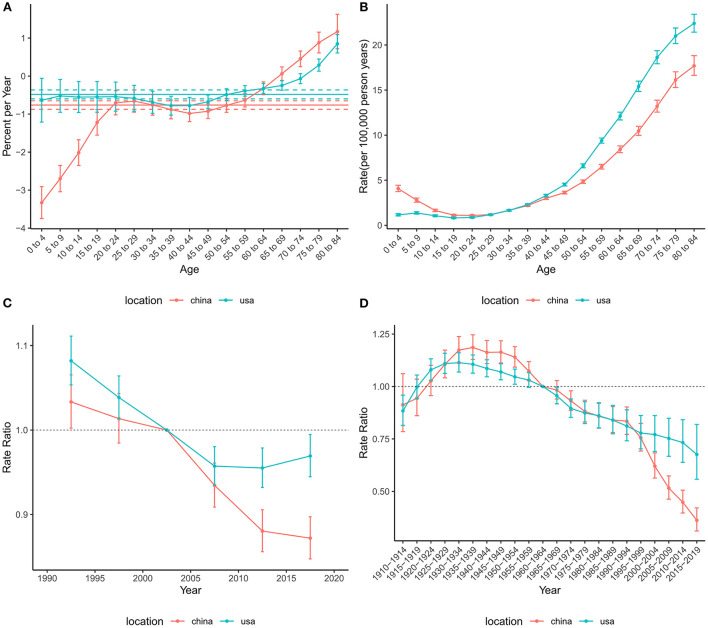
Age-period-cohort effects on the mortality rate of brain and CNS cancers in China and the US. **(A)** Net drift and local drift values for brain and CNS cancers mortality rate in China and the US. **(B)** Longitudinal age curves for brain and CNS cancers mortality in China and the US. **(C)** Period effects on the brain and CNS cancers mortality rate in China and the US relative to the reference period (2000–2004). **(D)** Cohort effects on the brain and CNS cancers mortality rate in China and the US relative to the reference cohort (1960–1964). Vertical solid bars represent the 95% confidence intervals. CNS, central nervous system.

The net drift for mortality across the study period was −0.76% (95% CI: −0.87% to −0.65%) in China and −0.48% (−0.60% to −0.36%) in the US (Figure 4), suggesting an overall decreasing trend in mortality risk in both countries. However, the local drift values for those older than 75 years were higher than 0, indicating that mortality in these populations has increased over the past 30 years. The overall risk of mortality showed an increasing trend in the US, while China tended to decline and then increase. The period effect showed that the risk of death decreased over time in China during the entire study period, while the risk in the US decreased before the 2010–2014 period and then increased slightly. The cohort RRs of mortality tended to rise and then fall in China and the US.

**Figure 5:**
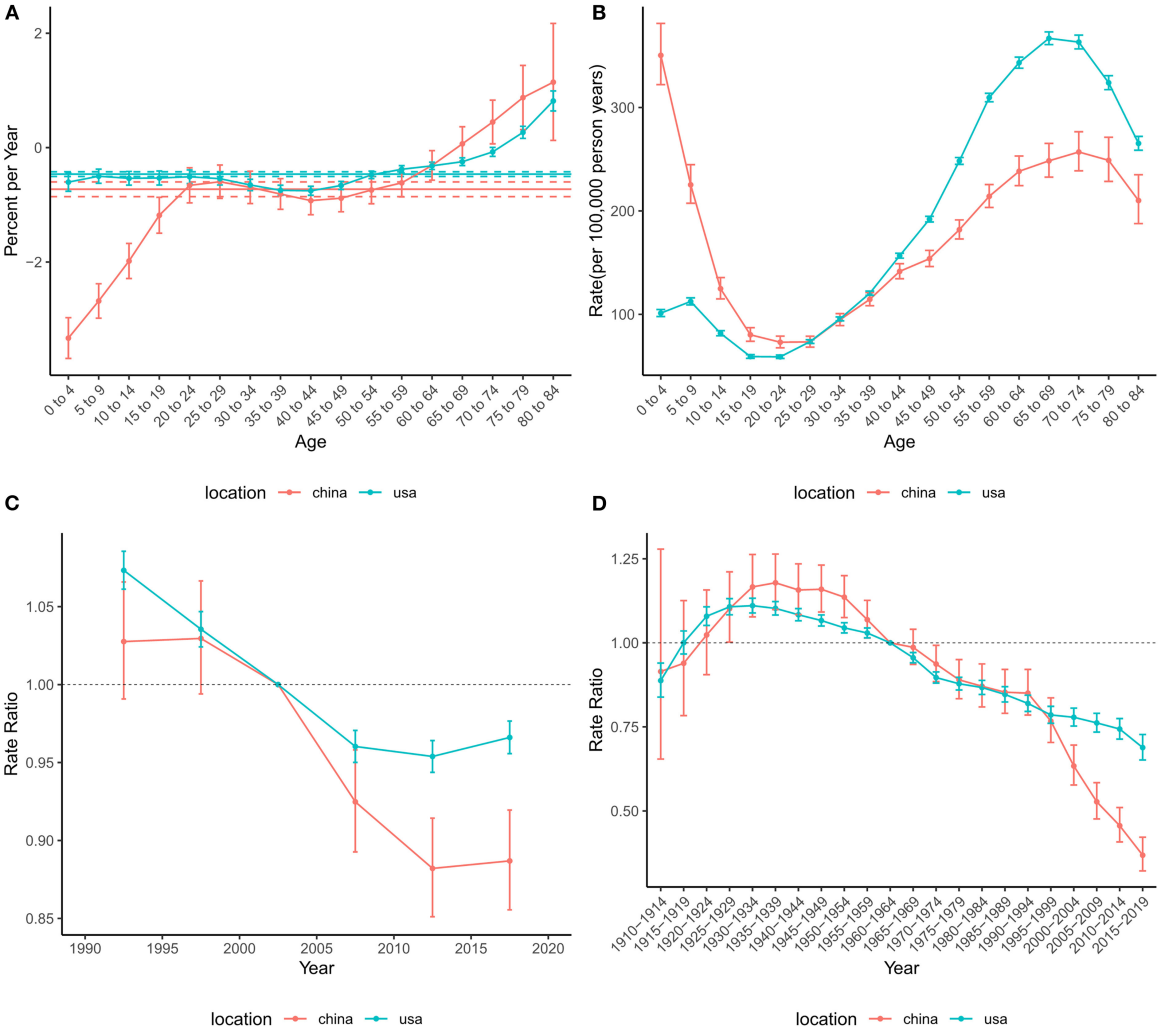
Age-period-cohort effects on the DALYs rate of brain and CNS cancers in China and the US. **(A)** Net drift and local drift values for brain and CNS cancers DALYs rate in China and the US. **(B)** Longitudinal age curves for the DALYs of brain and CNS cancers in China and the US. **(C)** Period effects on the DALYs rate of brain and CNS in China and the US relative to the reference period (2000–2004). **(D)** Cohort effects on the DALYs rate of brain and CNS cancers in China and the US relative to the reference cohort (1960–1964). Vertical solid bars represent the 95% confidence intervals. DALYs, disability-adjusted life-years; CNS, central nervous system.

Similar to mortality, the net drift values were negative in both China (−0.73%, 95%CI: −0.86% to −0.60%) and the US (−0.46%, 95%CI: −0.50% to −0.42%), indicating a decrease in the risk of DALYs during the study period (Figure 5). In addition, the local drifts aged higher than 75 were >0. The age effect on DALYs showed a bimodal distribution in both China and the US. The two peaks of age effects were in the 0–4 and 70–74 age groups in China, while those were in the 5–9 and 65–69 age groups in the US. The period effects in China and the United States declined significantly until the 2010–2014 period and then increased slightly. The overall trend for the cohort effect in China and the US showed an increase followed by a decline.

### Future trends in the burden of brain and CNS cancers from 2020 to 2030

**Figure 6:**
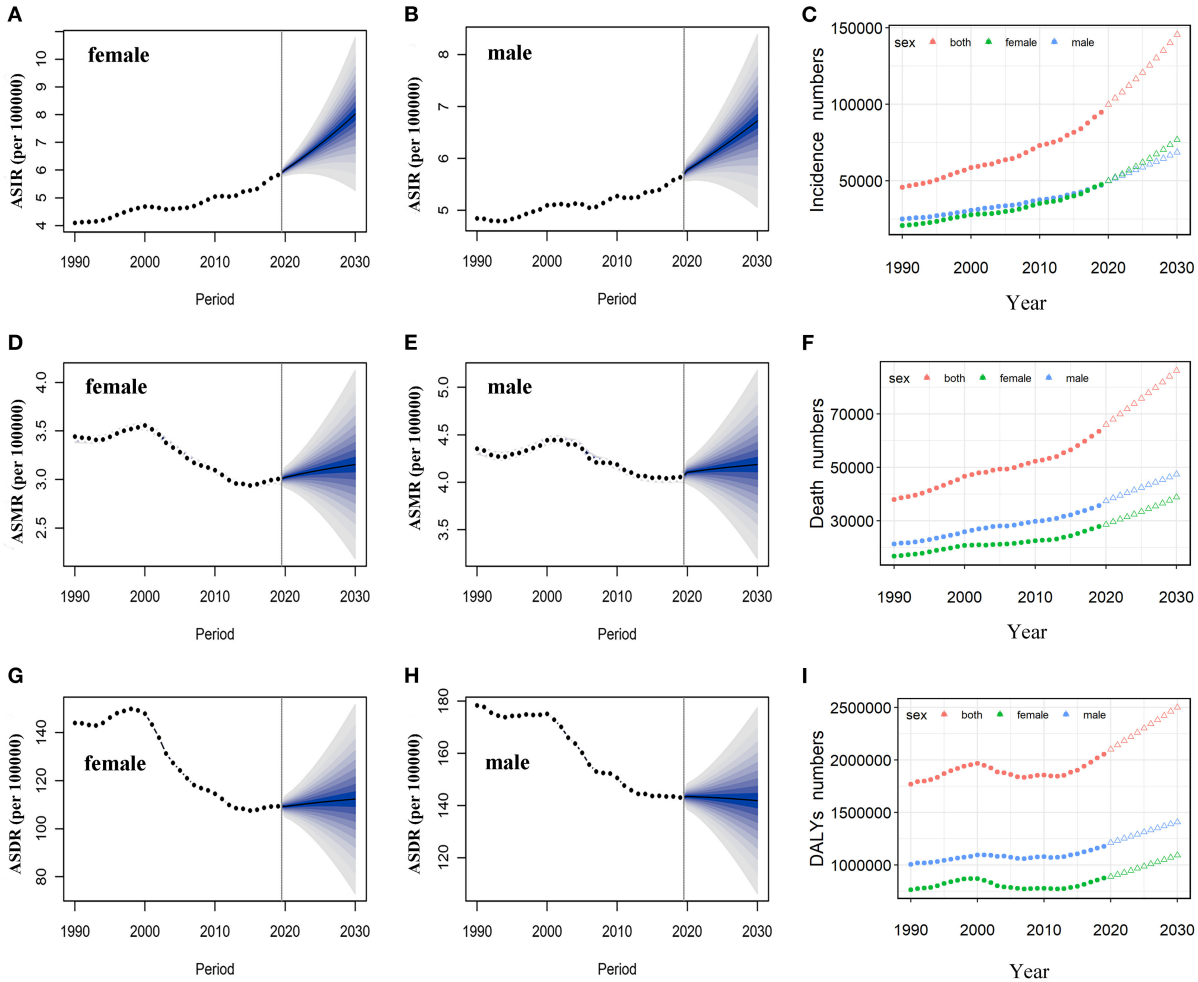
Temporal trends and projected ASIR, ASMR, and ASDR by sex, from 1990 to 2030 in China. Projected temporal trends in ASIR for Chinese women **(A)** and men **(B)** from 1990 to 2030. Projected temporal trends in ASMR for Chinese women **(D)** and men **(E)** from 1990 to 2030. Projected temporal trends in ASDR for Chinese women **(G)** and men **(H)** from 1990 to 2030. Projected numbers of incidences **(C)**, deaths **(F)**, and DALYs **(I)** from 1990 to 2030 in China. Observed (dashed lines) and predicted rates (solid lines). The fan shows the predictive distribution between the 5 and 95% quantiles. Observed (solid circle) and predicted numbers (triangle). DALYs, disability-adjusted life years; CNS, central nervous system; ASIR, age-standardized incidence rate; ASMR, age-standardized mortality rate; ASDR, age-standardized disability-adjusted life years rate.

**Figure 7:**
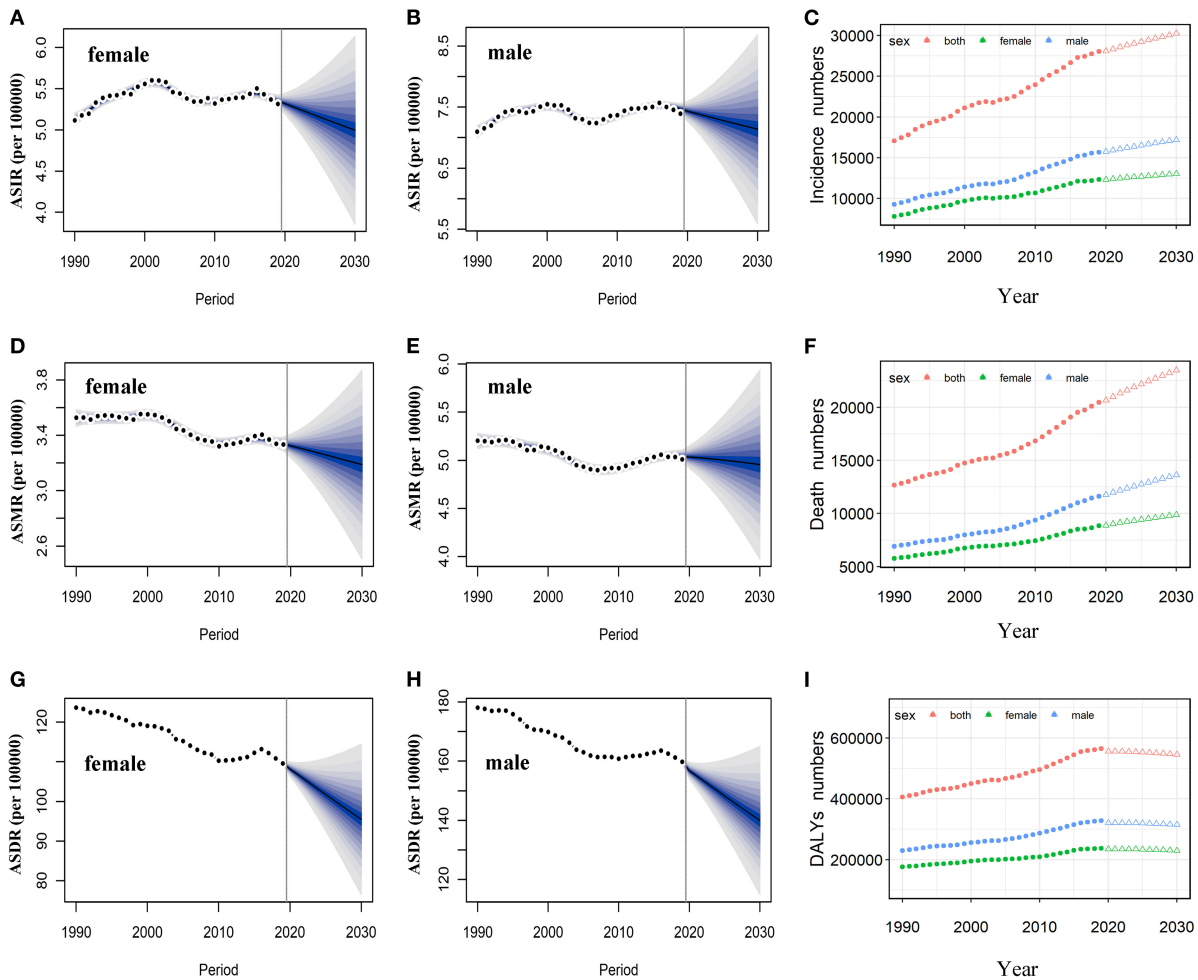
Temporal trends and projected ASIR, ASMR, and ASDR by sex, from 1990 to 2030 in the US. Projected temporal trends of ASIR for women **(A)** and men **(B)** in the US from 1990 to 2030. Projected temporal trends of ASMR for women **(D)** and men **(E)** in the US from 1990 to 2030. Projected temporal trends of ASDR for women **(G)** and men **(H)** in the US from 1990 to 2030. Projected numbers of incidences **(C)**, deaths **(F)**, and DALYs **(I)** from 1990 to 2030 in the US. Observed (dashed lines) and predicted rates (solid lines). The fan shows the predictive distribution between the 5 and 95% quantiles. Observed (solid circle) and predicted numbers (triangle). DALYs, disability-adjusted life years; CNS, central nervous system; ASIR, age-standardized incidence rate; ASMR, age-standardized mortality rate; ASDR, age-standardized disability-adjusted life years rate.

Based on the incidence and death data of brain and CNS cancers in China from 1990 to 2019 in the GBD database, we further predicted the burden of brain and CNS cancers in China in the next decade by applying the BAPC model. By 2030, the incidence cases of brain and CNS cancers were projected to increase to 145.65 thousand in China, with 68.73 thousand and 76.92 thousand cases for men and women, respectively (Table 2). We observed that the trend in ASIR for Chinese men and women would continue to increase over the upcoming decade (Figure 6). The ASIR in males and females was expected to reach 6.72/100,000 persons and 8.02/100,000 persons in 2030. Compared to China, the number of incidences in the United States was projected to increase slightly to 30.27 thousand in 2030, with 17.21 thousand and 13.06 thousand males and females, respectively. Moreover, the ASIR of both men and women in the United States showed a decreasing trend in the upcoming 10 years, with 7.14 per 100,000 persons and 5.0 per 100,000 for males and females in 2030, respectively (Figure 7). The children and elderly groups will continue to be the high-risk group for brain tumors in the next 10 years (Supplementary Figure 2).

**Table 2:** The predicted number of incidences, deaths, and DALYs for brain and CNS cancers in China and the US from 2020 to 2030.

	**China**				**The United States**			
	**2020 (*N*)**	**2030 (*N*)**	**Change (%) 2020–2030**	Change*(%) 1990–2030	**2020 (*N*)**	**2030 (*N*)**	**Change (%) 2020–2030**	Change*(%) 1990–2030
**Incidence**								
Both	99,977	145,645	45.68	217.68	28,064	30,268	7.85	77.26
Female	49,940	76,916	54.02	270.56	12,318	13,061	6.03	67.52
Male	50,037	68,729	37.36	173.93	15,745	17,207	9.29	85.44
**Death**								
Both	66,081	86,328	30.64	127.38	20,661	23,499	13.74	85.48
Female	28,623	38,851	35.73	132.50	8,886	9,884	11.23	71.49
Male	37,459	47,477	26.74	123.36	11,775	13,615	15.63	97.15
**DALYs**								
Both	2,102,263	2,501,151	18.97	41.34	555,908	545,454	−1.88	34.30
Female	889,728	1,093,767	22.93	43.20	234,902	230,139	−2.03	30.65
Male	1,212,535	1,407,384	16.07	39.92	321,006	315,316	−1.77	37.10

CNS, central nervous system; DALYs, disability-adjusted life years; N, number. *The number of cases in 1990 was derived from the observed number of GBD, while the cases in 2030 were projected numbers.

Overall, the number of deaths will continue to increase in both the United States and China. We projected that ~86.33 thousand persons will die attributed to brain and CNS cancers in 2030 in China, while only 23.5 thousand in the US (Table 2). In contrast to the past 30 years, we found that ASMR will increase in the next decade in China (Figure 6). The ASMR was projected to continue to be higher for Chinese men than women in 2030 (4.19 vs. 3.15 per 100,000 persons). Overall, the deaths caused by brain and CNS cancers in the US will continue to rise. However, the ASMR for men and women in the US will decline, it is predicted to fall to 4.95 per 100,000 persons and 3.19 per 100,000 persons, respectively, in 2030 (Figure 7). The elderly adults were more affected in terms of the number of deaths caused by brain and CNS cancers (Supplementary Figure 2).

The number of DALYs attributed to brain and CNS cancers was expected to increase to 2.5 million over the next 10 years, while the number in the US continues to decline to 545.45 thousand (Table 2). We found moderate increases in ASDR for Chinese women during the next decade, while men will continue to decrease (Figure 6). In addition, we observed a sharp decline in ASDR for both men and women in the US between 2020 and 2030 (Figure 7). The age distribution plot showed that DALYs caused by brain and CNS cancers were mainly concentrated in the children and elderly groups (Supplementary Figure 2).

## Discussion

In this study, we used the latest GBD (2019) data to conduct a comprehensive analysis of the disease burden of brain and CNS cancers and its temporal trends in China and the US by gender and age, and forecast the burden during 2020–2030. Our findings suggested that the current disease burden of brain and CNS cancers in China and the United States remained severe, primarily due to the increase in the number of incidences, deaths, and DALYs from 1990 to 2019, especially in China. Overall, ASIR showed an increasing trend in China and the US, while ASMR and ASDR showed a decreasing trend. Moreover, the increase in incidence and the decrease in mortality and DALYs were higher in China than in the US. Age distribution trends were similar in China and the United States, with concentrations in children and older adults. The risk of incidence increased over time in China, whereas the risk of death and DALYs decreased over time. The cohort effect showed an overall trend of increasing and then decreasing trend of burden in both China and the US. In addition, we projected that the burdens attributed to brain and CNS cancers will continue to rise in China over the next decade.

The American Cancer Society estimated 23,820 incident cases and 17,760 deaths in 2019 (28), while GBD estimates 28,021 incident cases (95% UI 21,371–33,113) and 20,459 deaths (95% UI 15,703–22,010) for the same year. National Cancer Center of China estimated 109,000 incident cases and 58,500 deaths in 2016 (29), while 84,094 incident cases (95% UI 65,590–99,975) and 58,213 deaths (95% UI 45,264–69,181) for the same year by GBD. Thus, the estimates by GBD 2019 were compatible with relevant cancer statistics research. The cases of incidence, deaths, and DALYs of brain and CNS cancers increased steadily from 1990 to 2019 in China and the US, implying a substantial burden remained in both countries. On the other hand, the rise in absolute numbers might be related to the increase in population and longer life expectancy (18).

Consistent with previous studies elsewhere (4, 7, 14), our study also found an increase in ASIR in China and the US over time. The increase in ASIR was more significant in China compared to the United States, which might be related to the growing aging population and improved imaging techniques in China (30, 31). In terms of sex, both sexes showed an upward trend from 1990 to 2019, with higher ASIR mainly in males. However, we found that the higher ASIR in females than in males since 2017 in China, with the highest increase. On the one hand, with increasing awareness of gender equality and growing attention to the rights of women, the increased accessibility of medical resources such as physical examinations, disease diagnosis, and treatment for Chinese women might have contributed to the rise in female incidence. On the other hand, the significant increase in female incidence implies that prevention and control to be strengthened for women in the future. Meanwhile, the ASMR and ASDR attributed to brain and CNS cancers declined during the study period in both countries, with a higher decrease for China. Early diagnosis of cancer, improved surgical procedures, better postoperative monitoring, and more effective routine use of chemotherapy might be associated with the declining trend for ASMR and ASDR (32, 33). Similar to other research findings (10, 34), we found that ASMR and ASDR were consistently higher in men than in women during the entire period, indicating that improvement in the prognosis of male patients was urgent.

With respect to age, the burden trends of brain and CNS cancers in the US and China showed a bimodal distribution, indicating that age was a risk factor for brain and CNS cancers. The two peaks were concentrated in childhood and older adults, indicating that prevention in children and treatment in the elderly should be strengthened. Similar to most cancer, the highest burden of brain and CNS cancers was concentrated in the elderly, which might be related to increased DNA damage and decline in physiological and immune function in the elderly (35, 36). Moreover, the higher mortality and DALYs in the elderly might be related to higher complications (37). Consistent with the CBTRUS report, our results also indicated that children are at high risk for brain tumors (10). There is no conclusive evidence on the causes of brain and CNS cancers in children, but the increase in the incidence of children might be related to maternal exposure to risk factors such as ionizing radiation, pesticides, and environmental pollution during pregnancy (38, 39). Childhood cancer survivors might suffer from long-term risks such as cognitive impairment due to the toxic effects of cranial irradiation, surgical treatment, and the toxicity of chemotherapeutic drugs (40, 41). Therefore, it is urgent to improve the surveillance and treatment of brain and CNS cancers in children.

The period effect of incidence showed a significant upward trend in China over time, while the change was modest in the US. The significant increase in incidence in China might be related to increased risk of exposure and the advancement of health services (42). Therefore, it is necessary to take measures against the cause of the disease to slow or reverse this trend in China, such as increased screening and reduced risk factor exposure. The period effect for mortality and DALYs declined significantly before the 2010–2014 period in China and the US. It is not difficult to find that the period effect in China has a clear decline trend after 2003. This might be related to the implementation of the New Rural Cooperative Medical System (NRCMS) in China since 2003, which has greatly improved access to medical services for rural residents in China (43). In addition, the improvement in treatment has contributed to this decline (44). However, the slight increase in the risk of death and DALYs in recent years might be related to the popularity of smartphones (45).

The cohort effect for incidence increased before 1990 and then decreased in China. In the early twentieth century, China was experiencing war, poor living conditions, and increased exposure to risk factors, which led to the increased risk of brain and CNS cancers. The gradual improvement in living and medical conditions since the post-1978 economic reforms in China contributed to the decline in incidence risk (46). The cohort risk of incidence in the US increased before 1925 and then tended to plateau. In the 1910s and early 1920s, ionizing radiation was used as a means of therapeutic abortion in the US. However, the expected termination of pregnancy did not occur in a substantial number of cases, resulting in full-term pregnancies (47). Exposure to ionizing radiation before birth would lead to the development of brain and CNS cancer. Overall, the cohort effect for death and DALYs showed an upward and then downward trend in China and the US. Early in life exposure to adverse risk factors has a huge impact on the future development of disease (48). Inferior medical care, poor education, and weak health awareness in early stages might have contributed to the high risk for death and DALYs in this early cohort (49, 50). The decreased risk of mortality and DALYs in the younger generations were possibly attributed to the fact that younger generations may receive better education and have a strong awareness of health and prevention of disease (49). On the other hand, improvements in medical treatment might facilitate the decline (33).

Overall, we predicted that the burden on the brain and CNS in China would continue to increase over the next 10 years. Moreover, we found the ASIR will continue to be higher for women than for men in the upcoming 10 years, it is urgent to strengthen the prevention and control of women. Compared to China, the burdens attributed to brain and CNS cancers in the US was expected to show a decreasing trend by sex in the next 10 years. However, the number of new cases and deaths in the US was projected to increase modestly during the following decade due to the growing population and changes in the aging population (51). In addition, we found that the ASMR and ASDR in China will increase modestly in the next 10 years, suggesting that further lessons from the prevention and control experience in the US are required to reverse this trend in the future.

However, our study had some limitations. First, GBD data were reconstructed from extensive data with different quality sources, resulting in potential bias from the actual data. Therefore, further validation based on a national epidemiological investigation was needed. Second, due to the lack of risk factor data for brain and CNS cancers in the GBD 2019 database, we were unable to investigate the effect of risk factors on cancer burden. The interpretations of the results need further confirmation. Third, there is heterogeneity across different histological subtypes of brain and CNS cancers. Important information might be obscure when analyzed together. However, the data on histological subtypes were unavailable in the GBD 2019 database, we were unable to analyze the cancer burden for different histological subtypes. Therefore, a comprehensive analysis by histologic subtype deserves further conduct.

## Conclusion

In conclusion, our study revealed that ASIR for brain and CNS cancers has increased over the past 30 years, despite some achievements in reducing mortality and DALYs attributable to brain and CNS cancers in China and the United States. The burden of disease attributable to brain tumors in the United States and China remained serious. Moreover, the burden of brain and CNS cancers will continue to increase over the upcoming decade in China. Therefore, we should strengthen the management and prevention of potential or known risk factors, focus on high-risk populations, improve diagnosis and treatment techniques, and reduce health care costs in subsequent public health policies for brain and CNS cancers.

## Data availability statement

The raw data of this study can be found through the Global Health Data Exchange Software (http://ghdx.healthdata.org/gbd-2019).

## Author contributions

JH and D-LL: study design. JH, HL, HY, F-XL, MT, and D-LL: data collection. JH: data analyses and manuscript writing. All authors: results interpretations. All authors contributed to the article and approved the submitted version.

## Funding

This study was supported by the National Natural Science Foundation of China (grant number: 82003522).

## Conflict of interest

The authors declare that the research was conducted in the absence of any commercial or financial relationships that could be construed as a potential conflict of interest.

## Publisher's note

All claims expressed in this article are solely those of the authors and do not necessarily represent those of their affiliated organizations, or those of the publisher, the editors and the reviewers. Any product that may be evaluated in this article, or claim that may be made by its manufacturer, is not guaranteed or endorsed by the publisher.
