# Supplementary material for: The comparative burden of brain and central nervous system cancers from 1990 to 2019 between China and the United States and predicting the future burden
Source: Front Public Health. 2022 Oct 21;10:1018836. doi: 10.3389/fpubh.2022.1018836 (PMC9635888; doi:10.3389/fpubh.2022.1018836)
Supplement: Supplementary file 2 [file Presentation_1.pdf]

Supplementary Material

1. Supplementary Figures

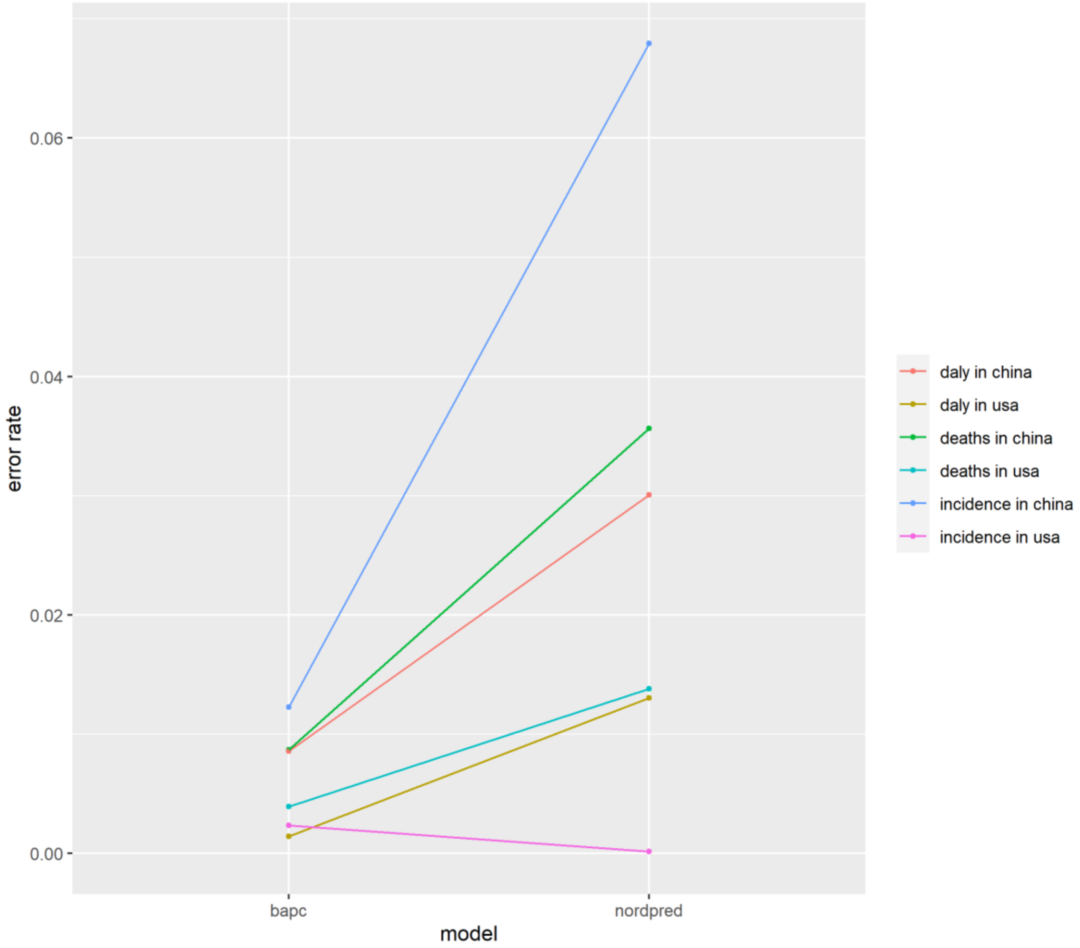

Supplementary Figure 1 Prediction error rates of Nordpred and BAPC models. Abbreviations: DALY: disability-adjusted life year; BAPC: Bayesian age-period-cohort.

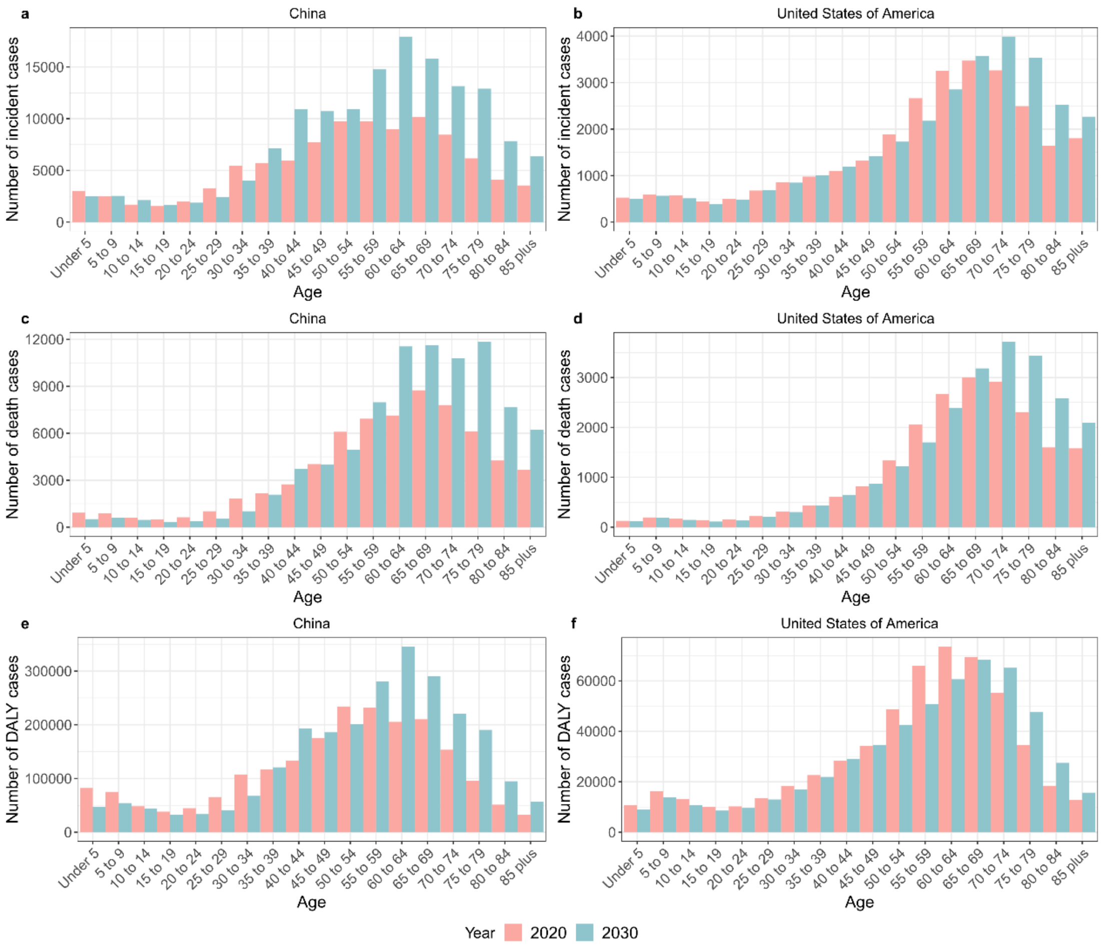

Supplementary Figure 2 Projected Incidence, Deaths, and DALYs for brain and CNS cancers by age group in China and the US in 2020 and 2030. Incidence numbers of brain and CNS cancers for each age group in China (a) and the US (b). Death numbers of brain and CNS cancers for each age group in China (c) and the US (d). DALYs numbers of brain and CNS cancers for each age group in China (e) and the US (f). Abbreviations: DALY: disability-adjusted life year; CNS, central nervous system.
